# Myocardial late gadolinium enhancement is associated with clinical presentation in Duchenne muscular dystrophy carriers

**DOI:** 10.1186/s12968-016-0281-y

**Published:** 2016-09-22

**Authors:** Paul Wexberg, Marion Avanzini, Julia Mascherbauer, Stefan Pfaffenberger, Birgit Freudenthaler, Reginald Bittner, Günther Bernert, Franz Weidinger

**Affiliations:** 1 2nd Medical Department, Krankenanstalt Rudolfstiftung, Vienna, Austria; 2 SVA-Gesundheitszentrum, Hartmanngasse 2b, Vienna, A-1051 Austria; 3 Division of Cardiology, Department Of Internal Medicine II, Medical University of Vienna, Vienna, Austria; 4 Neuromuscular Research Department, Center of Anatomy & Cell Biology, Medical University of Vienna, Vienna, Austria; 5 Gottfried von Preyer Children Hospital, Vienna, Austria

**Keywords:** Cardiovascular magnetic resonance, T1-mapping, Duchenne muscular dystrophy, Cardiomyopathy

## Abstract

**Background:**

Duchenne muscular dystrophy (DMD) is an X-linked recessive disease that occurs in males leading to immobility and death in early adulthood. Female carriers of DMD are generally asymptomatic, yet frequently develop dilated cardiomyopathy. This study aims to detect early cardiac manifestation in DMD using cardiovascular magnetic resonance (CMR) and to evaluate its association with clinical symptoms.

**Methods:**

Clinical assessment of DMD carriers included six minutes walk tests (6MWT), blood analysis, electrocardiography, echocardiography, and CMR using FLASH sequences to detect late gadolinium enhancement (LGE). T1-mapping using the Modified Look-Locker Inversion recovery (MOLLI) sequence was performed quantify extracellular volume (ECV).

**Results:**

Of 20 carriers (age 39.47 ± 12.96 years) 17 (89.5 %) were clinically asymptomatic. ECV was mildly elevated (29.79 ± 2.92 %) and LGE was detected in nine cases (45 %). LGE positive carriers had lower left ventricular ejection fraction in CMR (64.36 ± 5.78 vs. 56.67 ± 6.89 %, *p* = 0.014), higher bothCK (629.89 ± 317.48 vs. 256.18 ± 109.10 U/l, *p* = 0.002) and CK-MB (22.13 ± 5.25 vs. 12.11 ± 2.21 U/l, *p* = 0.001), as well as shorter walking distances during the 6MWT (432.44 ± 96.72 vs. 514.91 ± 66.80 m, *p* = 0.037). 90.9 % of subjects without LGE had normal pro-BNP, whereas in 66.7 % of those presenting LGE pro-BNP was elevated (*p* = 0.027). All individuals without LGE were in the NYHA class I, whereas all those in NYHA classes II and III showed positive for LGE (*p* = 0.066).

**Conclusions:**

Myocardial involvement shown as LGE in CMR occurs in a substantial number of DMD carriers; it is associated with clinical and morphometric signs of incipient heart failure. LGE is thus a sensitive parameter for the early diagnosis of cardiomyopathy in DMD carriers.

**Trial registration:**

Clinicaltrials.gov, NCT01712152 Trial registration: October 19, 2012.

First patient enrolled: September 27, 2012 (retrospectively registered).

## Background

Duchenne Muscular Dystrophy (DMD) is an X-linked recessive myodegenerative disease that is manifest in males from early childhood onwards [1, 2] owing to the absence or vast reduction of dystrophin. DMD patients suffer from progressive muscle weakness and are wheelchair bound by their teens. The majority of DMD patients die prematurely in their twenties. Involvement of the cardiac muscle is common, but often remains clinically silent for want of physical exertion. Corticosteroids have been found to delay loss of ambulation [3], but to date no cure has been found for the disease despite ongoing clinical trials.

Female carriers of DMD are often asymptomatic, but may also display skeletal muscle weakness and may have elevated CK blood levels. The exact prevalence is unknown, because most cases only come to light during diagnostic examinations related to a diseased family member. Given the longevity factor, myocardial involvement in carriers is clinically more relevant than in DMD patients: in 10 % of all carriers heart failure in the absence of skeletal muscle involvement is detected [4, 5], as compared to 1–2 % in the general population [6]. Furthermore, dystrophy of cardiomyocytes precedes the onset of clinical symptoms the result being that signs of cardiomyopathy (CMP) only come to the fore after significant cardiac function impairment has been confirmed. The American Academy of Paediatrics (AAP) thus recommends that every 5 years DMD-carriers undergo a cardiological examination that includes an ECG and echocardiography of DMD carriers every 5 years [5]. According to the guidelines issued by the European Society of Cardiology (ESC) medical therapy is indicated in cardiomyopathies of any origin in the event of symptoms or signs of heart failure. In DMD this refers basically to reduced left ventricular function (LVF) measured during echocardiography [6]. Standard medical treatment for heart failure has been successfully implemented in DMD patients [7–9], whereas data on the effectiveness of the same medication in DMD carriers are insufficient.

Cardiovascular magnetic resonance (CMR) permits non-invasive depiction of pathological changes in the myocardial structure such as fibrosis, oedema and fatty replacement of myocytes [10]. It has, thus, also been used to assess cardiac involvement in dystrophinopathies [11, 12]. DMD-patients display a diffuse distribution of intramyocardial fibrosis, as indicated by late gadolinium enhancement (LGE) on CMR [13]. There are some reports on DMD carriers with a similar pattern of disease even in absence of left ventricular dysfunction [14–16]. However, diffuse myocardial fibrosis often passes undetected during LGE analysis. Recent studies have proposed contrast-enhanced T1-mapping as a novel tool for determining diffuse fibrosis via CMR in non-ischemic cardiomyopathies [17–19].

Thus, the aim of our study was to: (i) assess the incidence of myocardial involvement in DMD-carriers using CMR; (ii) determine a pattern of focal and diffuse myocardial fibrosis; and (iii) evaluate a relationship with LVF and clinical condition.

## Methods

### Study population

DMD-carriers listed in the data base of the Gottfried von Preyer Children’s Hospital, an Austrian tertiary referral centre for neuromuscular diseases in children, were invited to participate in the study after giving written informed consent. Age, body weight and height were recored and the body surface area and body mass index were calculated. The patient’s medical records were used to determine the type of genetic mutation.

The inclusion criteria were: genetic and/or histological identification as a carrier of DMD; aged 18 years and above; provision in writing of informed consent; and a negative pregnancy test in women of childbearing potential. The exclusion criteria were: claustrophobia; excessive obesity to an extent that precluded performance of CMR; chronic renal failure with a GFR <30 ml/min/1,73 m^2^; implanted pacemakers/defibrillators or other conditions precluding the carriers from CMR assessment; severe arrhythmia; and inability to cooperate during CMR.

### Blood sampling

Prior to CMR, venous blood was drawn to determine blood count and blood chemistry, especially creatine-kinase (CK), creatine-kinase MB (CK-MB), creatinine, glomerular filtration rate and pro-brain natriuretic peptide (pro-BNP).

### Echocardiography

Echocardiography was performed using scanners (Vivid 7, GE Healthcare) with a 3.5-MHz transducer in standard imaging planes. Systolic LVF was measured using biplane Simpson’s method, while diastolic LVF was assessed by using the inflow signal at the mitral valve and by tissue Doppler imaging. Diastolic dysfunction was considered to be present if at least half of the following parameters exceeded their respective cut-off value: septal e’ < 7 cm/s, lateral e’ < 10 cm/s, average E/e’ ratio > 14, left atrium volume index > 34 ml/m^2^, tricuscpid regurgitation velocity > 2,8 m/s [20]. Valvular function was evaluated by means of 2D images and colour-coded Doppler.

### Cardiac magnetic resonance (CMR)

All patients underwent CMR examinations on a 1.5 T scanner (MAGNETOM Avanto, Siemens Healthcare GmbH, Erlangen, Germany). The tests comprised standard protocols including LGE (0.1 mmol/kg gadobutrol [Gadovist®; Bayer Vital GmbH, Leverkusen, Germany]) if the eGFR was >30 ml/min/1.73 m^2^. Left and right atrial volumes were assessed using the biplane area-length method. At the time of inserting the intravenous cannula, blood was drawn for the measurement of haematocrit and serum creatinine levels. LGE was measured on short axis stacks using a semi-automatic approach set at a threshold of five standard deviations above the mean signal intensity of healthy myocardium.

T1-mapping was performed using electrocardiographically triggered MOLLI based on a 5(3) 3 prototype (five acquisition heartbeats are followed by three recovery heartbeats and a further three acquisition heartbeats) on a short axis mid-cavity slice and a four-chamber view. This method generates an inline, pixel-based T1 map by acquiring a series of images over several heartbeats with shifted T1 times, inline motion correction and inline calculation of the T1 relaxation curve during one breath-hold. The T1 sequence parameters were: starting inversion time (TI) 120 ms; TI increment 80 ms; reconstructed matrix size 256 × 218; and measured matrix size 256 × 144 (phase encoding resolution 66 %, phase encoding field of view 85 %). T1 maps were created both before and 15 min after the application of the contrast agent. For post contrast T1-mapping, a 4(1) 3(1) 2 prototype was used. Regions of interest were defined as left ventricular myocardium without LGE in order to study the prognostic impact of diffuse extracellular matrix expansion that could not be detected during visual assessment. T1 values (ms) of the blood pool were obtained at a sufficient distance from the papillary muscles and the endomyocardial border. Mean T1 values from the short axis and the four-chamber view were calculated CMR-ECV was calculated using the formula [21]:

$$ \mathrm{C}\mathrm{M}\mathrm{R}\hbox{-} \mathrm{E}\mathrm{C}\mathrm{V}=\left(1-\mathrm{hematocrit}\right)\times \frac{\left(\frac{1}{\mathrm{T}1\ \mathrm{m}\mathrm{y}\mathrm{o}\ \mathrm{p}\mathrm{o}\mathrm{st}}\right)-\left(\frac{1}{\mathrm{T}1\ \mathrm{m}\mathrm{y}\mathrm{o}\ \mathrm{p}\mathrm{r}\mathrm{e}}\right)}{\left(\frac{1}{\mathrm{T}1\ \mathrm{blood}\ \mathrm{p}\mathrm{o}\mathrm{st}}\right)-\left(\frac{1}{\mathrm{T}1\ \mathrm{blood}\ \mathrm{p}\mathrm{r}\mathrm{e}}\right)} $$

where “T1 myo pre”/“T1 blood pre” indicates myocardial/blood native T1 times and “T1 myo post”/“T1 blood post” indicates T1 times of myocardium/blood 15 min after application of the contrast agent. ECV was considered normal ≤28.5 % [22]. We used a dedicated software (cmr42, Circle Cardiovascular Imaging Inc., Calgary, Alberta, Canada) for further CMR analysis.

The ventricular function was determined on short axis stacks using Simpson’s rule. The epicardial border of the left ventricle was traced in order to determine ventricular mass, multiplying the wall volume by the specific density of the myocardium (1,05 g/cm^3^).

### Study visits

At each visit blood was drawn to determine the parameters mentioned above and the patients underwent clinical examination. The clinical stage was recorded according to the New York Heart Association (NYHA) classification. Echo (P.W.) and CMR (B.F., S.P.) analyses were performed by experienced observers blinded to the pro-BNP values. All patients were scheduled for a follow-up examination 12 months later. If clinically indicated, additional follow-up examinations (clinical, echocardiography, CMR) were performed. In symptomatic patients with signs of myocardial involvement medical therapy of heart failure was initiated according to the ESC guidelines for the treatment of heart failure.

### Statistical analysis

Statistical analysis was performed using the SPSS package (SPSS 22, IBM Corp., Armonk, New York, USA). In normal distribution, data for continuous variables were expressed as mean ± standard deviation (SD) and intergroup comparisons were carried out using a two-sided unpaired Student’s *t*-test. Categorical parameters were compared using the chi-square test. Numeric changes over time were assessed by a paired *t*-test. Differences were considered statistically significant at a *p*-value < 0.05.

## Results

### Baseline

Twenty-two carriers agreed to participate in the study. CMR could not be performed in two carriers for reasons of claustrophobia; so they were excluded from the study. The demographic characteristics of the 20 subjects remaining (mean age 39.47 ± 12.96 years, range 21 to 62 years) are shown in Table 1. Seventeen of the carriers included (85.0 %) were clinically asymptomatic, two were in NYHA class II and one in NYHA III; the latter also displayed neurological symptoms (weakness of the thigh muscles).

**Table 1:** Clinical and imaging parameters by LGE

	LGE neg.	LGE pos.	*p*
Age (ys)	38.65 ± 10.84	41.56 ± 12.23	0.593
BMI (kg/m^2^)	23.84 ± 2.83	24.14 ± 5.45	0.442
6MWT (m)	514.91 ± 66.80	432.44 ± 96.72	0.037
RR syst (mmHg)	111.18 ± 11.29	127.11 ± 16.86	0.021
RR diast (mmHg)	75.00 ± 10.50	83.56 ± 14.37	0.141
HR (/min)	69.91 ± 11.76	69.67 ± 11.57	0.964
Hkt (%)	37.36 ± 4.42	39.32 ± 3.37	0.298
CK (U/l)	256.18 ± 109.10	629.89 ± 317.48	0.002
CK-MB (U/l)	12.11 ± 2.21	22.13 ± 5.25	0.001
Trop. T (ng/ml)	0.01 ± 0.02	0.02 ± 0.01	0.487
proBNP (ng/l)	80.70 ± 33.84	142.89 ± 93.35	0.088
EF Echo (%)	63.38 ± 7.03	59.75 ± 7.52	0.336
E/A	1.67 ± 0.57	3.91 ± 7.91	0.383
E/e’	5.21 ± 1.86	6.01 ± 3.59	0.548
IVS Echo (mm)	8.96 ± 0.96	10.44 ± 1.51	0.015
LVEF CMR (%)	64.36 ± 5.78	56.67 ± 6.89	0.014
RVEF CMR (%)	55.45 ± 12.52	57.11 ± 9.24	0.792
T1 native (ms)	1016.73 ± 72.06	994.89 ± 36.32	0.386
ECV (%)	30.77 ± 2.96	28.60 ± 2.55	0.500

*6MWT* Six minutes walk test, *BMI* body mass index, *BNP* brain-natriuretic peptide, *CK* creatine phosphokinase, *ECV* extracellular volume, *EF Echo* ejection fraction determined by echocardiography, *IVS* intraventricular septum determined by echocardiography, *LGE* late gadolinium enhancement, *LVEF CMR* left-ventricular ejection fraction determined by cardiovascular magnetic resonance, *RR syst* systolic blood pressure, *RR diast* diastolic blood pressure, *RVEF CMR* right-ventricular ejection fraction determined by cardiac magnetic resonance, *Trop. T* troponin T, *T1 native* native T1-time of the myocardium

Overall, mean ECV was mildly elevated (29.79 ± 2.92 %) with abnormal values in 65.0 % of all cases. These subjects did not differ from those with normal values in regard to clinical and laboratory parameters of cardiac function (data not shown). In nine carriers (45.0 %) LGE was visible in the myocardium of the left ventricle. This group presented higher septum thickness and lower EF in CMR, had higher systolic blood pressure, higher blood levels of CK and CK-MB and shorter walking distances at 6MWT. 90.9 % (*n* = 10) of the subjects without LGE had normal pro-BNP, whereas 66.7 % (*n* = 6) of those with LGE had elevated pro-BNP values (*p* = 0.027). Pro-BNP tended to be higher as well. As a marker of diffuse myocardial fibrosis ECV did not differ significantly between patients with and without LGE (Table 1).

Comparing dyspnea, all 11 individuals without LGE were in NYHA class I, whereas in all subjects in NYHA II (*n* = 2) and III (*n* = 1) LGE was present (*p* = 0.066).

Dividing the cohort in those below (*n* = 13 [65.0 %]) and those above (*n* = 7 [25.0 %]) the age-corrected cut-off value of pro-BNP for females, the latter had higher CK and CK-MB levels and shorter distances covered during the 6MWT (421.29 ± 105.20 vs. 508.23 ± 66.03 m, *p* = 0.035). Septum thickness was higher and ejection fraction in CMR lower. Systolic blood pressure and ECV were comparable (Table 2). LGE was present in 85.7 % of subjects with elevated pro-BNP compared to 23.1 % in those with normal levels (*p* = 0.027).

**Table 2:** Clinical and imaging parameters by pro-BNP

	low pro-BNP	high pro-BNP	*p*
Age (ys)	38.04 ± 10.75	43.39 ± 12.19	0.324
BMI (kg/m^2^)	24.6 ± 4.72	22.81 ± 2.46	0.139
6MWT (m)	508.23 ± 66.03	421.29 ± 105.20	0.035
RR syst (mmHg)	117.69 ± 19.12	119.57 ± 8.14	0.809
RR diast (mmHg)	80.54 ± 14.31	75.71 ± 9.60	0.437
HR (/min)	70.08 ± 11.81	69.29 ± 11.37	0.887
Hkt (%)	37.25 ± 4.76	39.59 ± 2.42	0.301
CK (U/l)	323.08 ± 192.54	612.43 ± 363.06	0.030
CK-MB (U/l)	13.45 ± 3.14	23.00 ± 6.36	0.001
Trop. T (ng/ml)	0.01 ± 0.02	0.02 ± 0.01	0.392
EF Echo (%)	61.60 ± 5.58	61.5 ± 10.13	0.980
E/A	3.65 ± 6.74	1.16 ± 0.35	0.348
E/e’	5.47 ± 2.28	5.77 ± 3.66	0.832
IVS Echo (mm)	9.04 ± 1.23	10.71 ± 1.11	0.008
LVEF CMR (%)	63.54 ± 5.95	56.00 ± 7.35	0.023
RVEF CMR (%)	56.46 ± 5.61	56.00 ± 5.57	0.085
T1 native (ms)	1014.69 ± 64.00	992.43 ± 43.39	0.370
ECV (%)	30.03 ± 2.97	29.36 ± 3.02	0.642

*6MWT* Six minutes walk test, *BMI* body mass index, *BNP* brain-natriuretic peptide, *CK* creatine phosphokinase, *ECV* extracellular volume, *EF Echo* ejection fraction determined by echocardiography, *IVS* intraventricular septum determined by echocardiography, *LGE* late gadolinium enhancement, *LVEF CMR* left-ventricular ejection fraction determined by cardiovascular magnetic resonance, *RR syst* systolic blood pressure, *RR diast*, diastolic blood pressure, *RVEF CMR*, right-ventricular ejection fraction determined by cardiovascular magnetic resonance, *Trop. T* troponin T, *T1 native* native T1-time of the myocardium

The genetic analysis and the distribution of LGE are shown in Table 3.

**Table 3:** Mutations and presence of LGE and LVEF

Patient	Age (ys)	Mutation	LGE	LGE location	LGE distribution	# segments	LVEF (%)
1	42	del. exon 12–29	+	inf, lat	end, mid	3	54
2	40	del. 48–50	-	-	-	0	58
3	38	point mutation intron 40	-	-	-	0	72
4	40	exon 48 (somatic mosaic)	-	-	-	0	70
5	24	n. k.	+	inf, lat	mid	6	42
6	26	dupl. exon 3–34	+	inf, lat	mid	2	58
7	62	n. k.	+	inf, lat	mid	4	64
8	38	nonsense mutation exon 12	+	lat	mid	1	65
9	55	n. k.	+	ant, lat	mid	6	57
10	39	n. k.	+	lat	epi	2	53
11	41	n. k.	-	-	-	0	61
12	29	dupl. exon 1–2 and 39–34	-	-	-	0	66
13	59	dupl. exon 1–2 and 39–34	-	-	-	0	74
14	22	dupl. exon 1–2 and 39–34	-	-	-	0	59
15	40	dupl. exon 1–2 and 39–34	-	-	-	0	61
16	34	del. exon 23	-	-	-	0	57
17	41	point mutation exon 24	+	lat	mid, epi	3	61
18	29	del. exon 44	-	-	-	0	66
19	53	del. exon 1–11	-	-	-	0	64
20	47	del. exon 43	+	ant, lat	mid	2	56

*ant* anterior, *del.* deletion, *dupl.* duplication, *end* subendocardial, *epi* subepicardial, *inf* inferior, *lat* lateral, *LGE* late gadolinium enhancement, *LVEF* left ventricular ejection fraction (measured by CMR), *mid* midmyocardial, *n. k.* not known, *# segments* number of left ventricular segments involved

### Follow-up

All subjects were alive 12 months after the initial visit and no major adverse cardiac events were reported. Only eight subjects came to the scheduled follow-up examination, seven of whom had had no LGE visible at the initial CMR examination. The main reasons given for dropping out of the study were social in origin, relating to caring for the diseased child and psychological distress following a son’s death. The data, thus, do not suffice for purposes of statistical analysis. However, none of the women with negative CMR developed LGE during the follow-up period. Overall, no substantial clinically relevant deterioration was observed and ECV remained practically unchanged (−1,84 ± 1.81 %); the subject diagnosed with LGE during the initial examination (#1) walked a shorter distance during the 6MWT conducted at the follow-up examination (163 vs. 210 m), but no change was detected in terms of pro-BNP, LVF or ECV.

## Discussion

Our study showed that ECV is elevated in DMD carriers; and that LGE is associated with LVF and shorter distances covered during the 6MWT.

Reviewing the literature, very limited data exists on the natural history of carriers of DMD. Prognostic predictors are therefore difficult to detect. In general, a broad variety of symptoms is to be found in female conductors, which, in part, can be explained by chromosome X inactivation [23, 24]. In general the clinical presentation is much less dramatic than in male patients. In a cohort study performed in the Netherlands 22 % of the carriers displayed muscle weakness of varying severity, eight per cent had dilated cardiomyopathy and asymptomatic dilatation of the left ventricle was present in 18 % [25]. CMR studies indicate that fibrosis is involved in myocardial involvement in dystrophinopathies [13–16]; this appears to be a consequence of the same mechanisms observed in skeletal muscles [25]: Cardiomyocyte membranes are disrupted owing to the lack of dystrophin and a subsequent increase in intracellular concentration of calcium [26]. That leads to an activation of calcium-dependent proteases resulting in changes to the muscle cell membrane, the cycoskeleton and myofibrillae and ultimately to cell necrosis [27–30]. The increase in endo- and perimysial connective tissue leads to progressive fibrotic degeneration [31, 32] and clinically to the typical inevitable immobilisation and demise of the patients.

In our cohort 45 % of the carriers displayed morphological signs of myocardial involvement. While the detection of LGE and the influence on systolic LVF is in accordance with findings in recent publications [33–36] an association with clinical presentation has not been shown yet [36]. We found a statistically significant difference in cardiovascular and musculoskeletal parameters such as contraction of the distance walked during the 6MWT and higher blood levels of CK and CK-MB, as well as a trend towards higher pro-BNP levels and higher NYHA class in the presence of LGE. Systolic blood pressure was also higher in this particular group, so that one might speculate as to a haemodynamic confounding effect. However, only two of the subjects in the group with LGE (#8, #20) had hypertension (160/110 mmHg and 147/102 mmHg, respectively) further to which when classifying the probands according to their pro-BNP values blood pressure levels were comparable. Thus, our findings tend to suggest a correlation between the extent of myocardial involvement and clinical presentation.

Given the underlying pathogenetic process myocardial fibrosis is a dynamic process. A recent longitudinal study of 98 DMD-patients with at least four CMR assessments established that 52 % developed LGE during the observation period. The decline in LVF was more pronounced once LGE had developed [37]. It appears justified to speculate that a similar development might occur in DMD-carriers. On account of the broad variety of mutations, we could not detect any correlation between the presence of LGE and a specific genotype. Within one family with the same mutation (duplication of exons 1–2 and 39–34) LGE was not present in either youngest member (aged 21) or the oldest (aged 59), however, our cohort was too small to deduce a clear association.

**Fig. 1:**
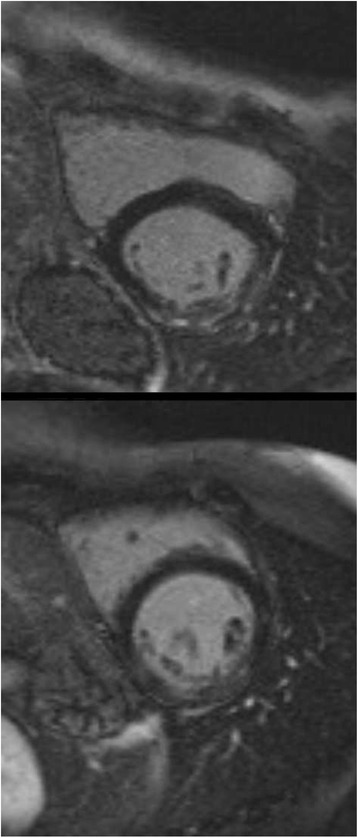
Diffuse myocardial fibrosis. Short-axis view (inversion-recovery gradient-echo) depicting diffuse myocardial fibrosis in two carriers of DMD. Note the patchy pattern of LGE along the lateral and inferolateral wall

Owing to their longevity DMD-carriers often develop heart failure in the absence of classical causes such as ischemic or inflammatory heart disease. According to our data, diastolic dysfunction does not appear to be associated with LGE according to our data; this is borne out by the findings of others [35]. Screening for morphological signs might thus help to detect subjects who are at risk in terms of developing cardiomyopathy. Given the predictive value of myocardial fibrosis [22, 37, 38], it is quite conceivable that the early initiation of neurohumoral therapy, the gold standard in the treatment of heart failure [6], might help to prevent LVF deterioration. Myocardial fibrosis can either be focal, as shown by LGE, or diffuse, as represented by an elevated ECV. In our cohort we found focal areas of mid-myocardial LGE which, for the most part, were located in predominantly lateral and inferolateral wall segments (Table 3, Fig. 1), thus confirming earlier reports [33–36]. At the same time CMR T1-mapping also revealed a generally elevated mean ECV. One might speculate that: (i) in general, DMD carriers may display diffusely of focally distributed myocardial fibrosis; or (ii) LGE is an advanced form of initially diffuse fibrosis. However, the data do not suffice to support fully this hypothesis.

### Study limitations

DMD is a rare disease; its carriers are regularly detected either fortuitously or in the course of a diseased relative being examined. In addition, the women so affected have to bear the burden of witnessing their sons as they progressively waste away and the responsibility of providing ever-increasing care. Thus, the number of subjects who were willing to participate fully in our study was low. In particular, it proved impossible in most cases to conduct the follow-up examinations scheduled for 12 months later. Such a small cohort also excluded special statistical applications (e.g. multivariate analysis) that would have permitted correction for confounders.

A larger cohort might have also provided a a firmer basis on which to assess a possible correlation between genotype and phenotype. In five carriers no mutation was detected; however, given the histological confirmation of DMD those carriers underwent further analysis.

Progression of myocardial fibrosis might be a slow process and a longer clinical follow-up might be necessary as to permit a prognostic interpretation of our findings and allow for the dynamic changes in diffuse and focal presentations.

To date, no precise normative values have been determined for ECV. However, on applying the highest tertile of Wong et al. [22] we have recently been able to demonstrate a prognostic impact of ECV [38]. That cut-off value was therefore applied throughout the present study.

## Conclusions

On using CMR, a substantial proportion of the DMD-carriers examined displayed signs of myocardial fibrosis which is associated with clinical parameters. Screening for early morphological signs of myocardial involvement may be potentially useful in the detection of subjects at risk of developing heart failure. CMR with an LGE-study should be performed during the diagnostic examination of this cohort. In further studies, the longitudinal development of myocardial fibrosis should be evaluated. It remains to be clarified in long-term studies whether the presence of LGE is an indication of the need to start specific neurohumoral medication.

## Abbreviations

6MWT: Six-minute walk test
BNP: Brain natriuretic peptide
CK: Creatine-kinase
CMR: Cardiovascular magnetic resonance
DMD: Duchenne muscular dystrophy
ECV: Extracellular volume
EF: Ejection fraction
FLASH: Fast low-angle shot
GFR: Glomerular filtration rate
LGE: Late gadolinium enhancement
LVF: Left ventricular function
MOLLI: Modified look-locker inversion recovery
NYHA: New York Heart Association
